# Evaluation of Therapeutic Vancomycin Monitoring in Taiwan

**DOI:** 10.1128/spectrum.01562-21

**Published:** 2022-04-12

**Authors:** Tzu-Ting Chen, Meng-Pu Liu, Hsing-Chih Sun

**Affiliations:** a Department of Pharmacy, Taipei Medical University Hospital, Taipei Medical University, Taipei, Taiwan; b Graduate Institute of Cancer Biology and Drug Discovery, Taipei Medical University, Taipei, Taiwan; Emory University School of Medicine

**Keywords:** acute kidney injury (AKI), area under the concentration–time curve (AUC), minimum inhibitory concentration (MIC), methicillin-resistant *Staphylococcus aureus* (MRSA), pharmacodynamics, therapeutic drug monitoring (TDM), vancomycin

## Abstract

This study aimed to evaluate whether trough level-guided monitoring can be replaced by area under the concentration–time curve (AUC) and MIC ratio-guided monitoring (AUC/MIC ratio = 400) in patients infected with methicillin-resistant Staphylococcus aureus (MRSA) with a vancomycin MIC = 1 mg/L in Taiwan. In this retrospective study, patients treated with vancomycin for Methicillin-resistant Staphylococcus aureus (MRSA) infection were recruited from a teaching hospital in Taiwan from January 2016 to December 2017. Average trough concentrations were adjusted based on the average daily vancomycin dose, and the AUC/MIC ratio was calculated using the AUC/MIC conversion formula to analyze the correlation between trough or AUC/MIC ratio, nephrotoxicity, and clinical efficacy. As the primary outcome, the overall mean adjusted vancomycin average AUC/MIC ratio was 526.87 for a total of 102 patients. A total of 67% and 76% of the patients attained an AUC/MIC of ≥400 when the adjusted vancomycin trough concentrations were 10 to 15 mg/L and 15 to 20 mg/L, respectively. Additionally, 81.37% of the total study population had MRSA isolates with a vancomycin MIC of ≤1 mg/L. Moreover, in the subgroup, 92% of the patients attained an AUC/MIC of ≥400 on receiving vancomycin in the 10 to 15 mg/L trough range. An AUC/MIC of ≥400 was attained in patients infected with MRSA strains, who were treated by maintaining the vancomycin trough concentrations at 10 to 15 mg/L. Moreover, these patients demonstrated a lower incidence of nephrotoxicity. These findings support the use of the AUC/MIC ratio as a useful marker for the therapeutic monitoring of vancomycin owing to the clinical efficacy and safety of vancomycin in Taiwan.

**IMPORTANCE** Since 2020, the Infectious Diseases Society of America (IDSA) updated vancomycin guidelines, and vancomycin AUC therapeutic drug monitor was updated to AUC/MIC in the United States. But acceptable rate of infection physicians in Taiwan was low. That is why this study evaluated in Taiwan.

## INTRODUCTION

Vancomycin has always been the first choice of treatment for Methicillin-resistant Staphylococcus aureus (MRSA) infection (1–3). The dosage of vancomycin is calculated based on body weight, and its concentration needs to be monitored to maintain the serum trough concentrations (troughs) at 10 to 20 mg/L. In 2009, the Infectious Diseases Society of America (IDSA) suggested that vancomycin trough concentration is an indicator of the relatively accurate and feasible monitoring of its efficacy (1). In 2020, the IDSA released updated guidelines suggesting that the 24 h area under the concentration–time curve (AUC) divided by the MIC is an indicator for the safe and accurate monitoring (target range: 400 to 600), which caused a massive change in global pharmaceutical care (2).

Existing studies have proved that maintaining high vancomycin trough concentrations (15 to 20 mg/L) is not positively correlated with treatment success (4); there are also reports suggesting that MRSA strains with a high MIC are correlated with unsuccessful treatment of infections (5). Although the clinical efficacy and safety of vancomycin with AUC/MIC of ≥400 are yet to be proved, some studies proposed that maintaining a high AUC/MIC ratio can significantly lower the incidence of death and treatment failure (6). In addition, in 2014, Neely suggested that for patients infected with bacterial strains having vancomycin MIC = 1 mg/L, approximately 50% attained an AUC/MIC of ≥400 without the need of the vancomycin trough concentration reaching 15 to 20 mg/L (7).

It has been acknowledged that if vancomycin MIC is ≥1 mg/L, higher trough concentrations of vancomycin (>15 mg/L) are needed to attain an AUC/MIC = 400, which may increase the risk of acute kidney injury (AKI) (4, 5). Other risk factors include the concomitant use of other antibiotics or nephrotoxic medications (e.g., nonsteroidal anti-inflammatory drugs), prolonged hospitalization duration, higher costs of medical care, and increased mortality rate (4, 8). According to IDSA’s latest guidelines released in 2020, the United States has already adopted AUC/MIC ratio as an indicator of therapeutic monitoring of vancomycin; however, studies on relevant pharmacodynamics and AUC/MIC conversion are yet to be published in Taiwan. In this retrospective study, we aimed to evaluate whether vancomycin trough concentrations of 10 to 15 mg/L were associated with increased attainment of AUC/MIC of ≥400 in patients with suspected MRSA infection in Taiwan.

## RESULTS

### Study population and vancomycin dosage characteristics.

**FIG 1:**
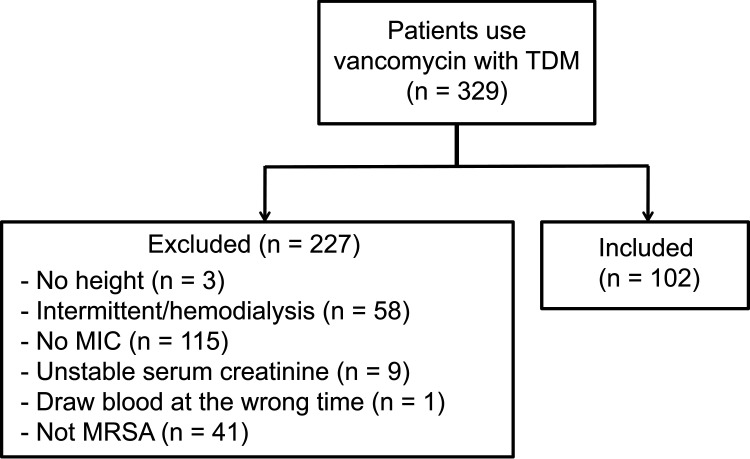
Patient inclusion criteria in this study. TDM, therapeutic drug monitoring; MIC, minimum inhibitory concentration; MRSA, methicillin-resistant Staphylococcus aureus.

Between January 2016 and December 2017, a total of 329 patients receiving vancomycin were identified. Of these, 227 patients were excluded due to one of the following reasons: unknown MIC, unstable renal function, unknown height, no MRSA infections, inappropriate time of blood sample collection, and/or undergoing hemodialysis. A total of 102 patients were included in the study (Fig. 1). Patient features, ward type, and analyzed vancomycin dosing characteristics, such as the drug dose, MIC value, and derived AUC/MIC results, are provided in Table 1. The included patients had an average age of 66.5 years, with 25.49% admitted to the intensive care unit. A total of 81.37% of the patients were infected with MRSA strains with a vancomycin MIC of ≤1 mg/L. The top four MRSA isolates were obtained from the samples of blood in 50% of the patients, followed by that of sputum (13.73%), wound (12.75%), and pus (12.75%) (Table 2). An AUC/MIC of 1,015 mg/L was considered as the reference value in the control group for comparison as well as the comparative endpoint of this study.

**TABLE 1:** Baseline demographics and adjusted vancomycin dosing characteristics^*a*^

Characteristic	Original vancomycin dose group (*n* = 102)		Adjusted vancomycin daily dose group (*n* = 102)	
Mean age, yrs (SD)	66.54 ± 18.25			
Sex (%) Male Female	59 (57.84) 43 (42.16)			
ICU setting (%)	26 (25.49)			
Non-ICU setting (%)	76 (74.51)			
Mean Scr, mg/dL (IQR)	0.9 (0.6–0.9)			
Mean ClCr, mL/min (IQR)	77.81 (40.34–90.27)			
Mean wt, kg (IQR)	60.7 (49.4–70)		55.43 (47.6–62.87)	
Mean wt-based dose, mg/kg (SD)	13.4 ± 4.64			
Mean wt-based daily dose, mg/kg/day (SD)	27.07 ± 12.59		30.04 ± 13.95	
Mean daily dose, mg/day (SD)	1634.8 ± 810.41		1675.51 ± 840.80	
Mean AUC, mg × hr/L (SD)	579.45 ± 306.61		579.39 ± 281.54	
MIC (%) MIC = 0.5 MIC = 1 MIC = 2	1 (0.98) 82 (80.39) 19 (18.63)			
Mean AUC/MIC ratio (IQR)	530.72 (318.46 to 648.43)		526.87 (371.82 to 628.19)	
AUC/MIC (%)	< 400 (37.25%)	≥ 400 (62.75%)	< 400 (34.31%)	≥ 400 (65.69%)
Trough < 10 mg/L Trough 10–15 mg/L Trough 15–20 mg/L Trough > 20 mg/L	23 9 5 1	19 25 9 11	19 7 7 2	17 22 14 14

a SD, standard deviation; IQR, interquartile range; ICU, intensive care unit; Scr, serum creatinine; ClCr, creatinine clearance; AUC, area under the concentration–time curve; MIC, minimum inhibitory concentration.

**TABLE 2:** Site of MRSA growth (*n* = 102)^*a*^

In culture	Value (%)
Sputum	14 (13.73%)
Wound	13 (12.75%)
Blood	51 (50%)
Urine	3 (2.94%)
Pleural fluid	4 (3.92%)
Ascites	2 (1.96%)
Pus	13 (12.75%)
Tip	1 (0.98%)
Biopsy tissues	1 (0.98%)

a MRSA, Methicillin-resistant Staphylococcus aureus; % of MRSA isolates identified from each site.

### Primary outcome.

The overall mean AUC/MIC was 530.72, with an AUC/MIC ratio of 526.87 adjusted using the average daily dose of vancomycin. A total of 67 of 102 patients (65.69%) attained an AUC/MIC of ≥400 as shown in Table 1.

**FIG 2:**
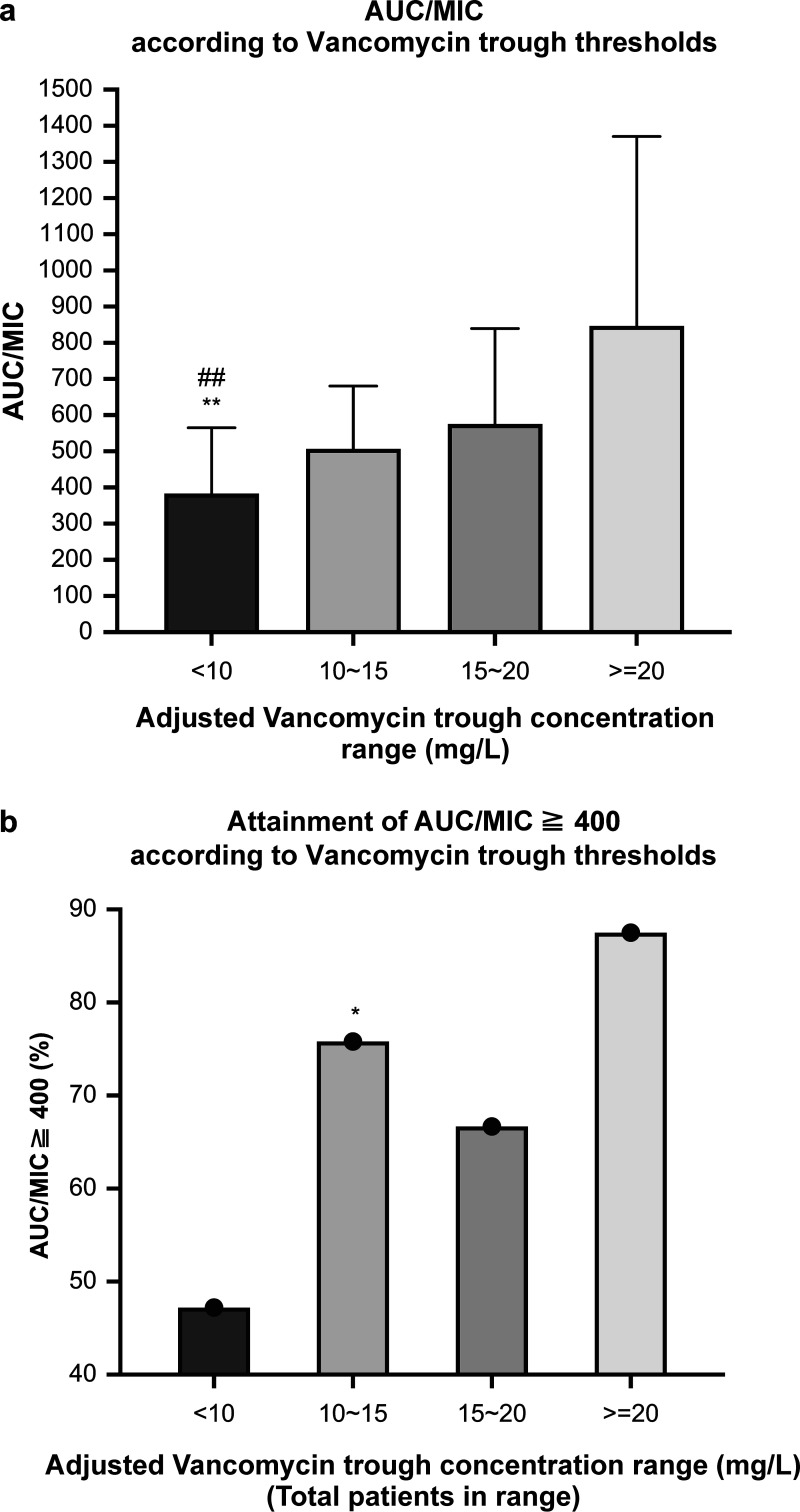
(a) Distribution of an AUC/MIC of ≥400 in the study population of 102 patients according to the four different adjusted vancomycin trough ranges. **, Indicates statistical significance when compared to the reference trough range of 10 to 15 mg/L in terms of attaining an AUC/MIC of ≥400. The Kruskal–Wallis test, followed by multiple comparison tests were used to analyze the data. The median values were significant (*P* < 0.05). ^##^, Indicates statistical significance when compared with the reference trough range of 10–15 mg/L in terms of attaining an AUC/MIC of ≥400. (b) Attainment of an AUC/MIC of ≥400 distribution according to the four different adjusted vancomycin trough ranges. *, Indicates statistical significance when compared with the reference trough range of 10 to 15 mg/L in terms of attaining an AUC/MIC of ≥400. AUC, area under the concentration–time curve; MIC, minimum inhibitory concentration.

After the trough concentrations were classified into four trough ranges based on the adjusted vancomycin troughs, the study found that the percentage of patients in the 10 to 15 mg/L and 15 to 20 mg/L trough ranges that attained an AUC/MIC of ≥400 were 76% and 67%, respectively. As represented in Figure 2a, a trough of <10 mg/L was significantly associated with a decreased likelihood of attaining an AUC/MIC of ≥400, whereas this likelihood was much more in high trough concentrations of ≥10 to 15 mg/L (Kruskal–Wallis test). In the study population of 102 patients, 76% of patients in the 10 to 15 mg/L trough range attained the AUC/MIC of ≥400, which was 9% higher than the percentage of those in the 15 to 20 mg/L trough range. The difference between the percentage of patients in the 10 to 15 mg/L trough range attaining AUC/MIC of ≥400 and that in the <10 mg/L trough range was statistically significant (Mann–Whitney U test [two-tailed] was performed for nonparametric data with a significant difference of *P* = 0.002) (Fig. 2b).

### Secondary analysis based on subgroups.

**FIG 3:**
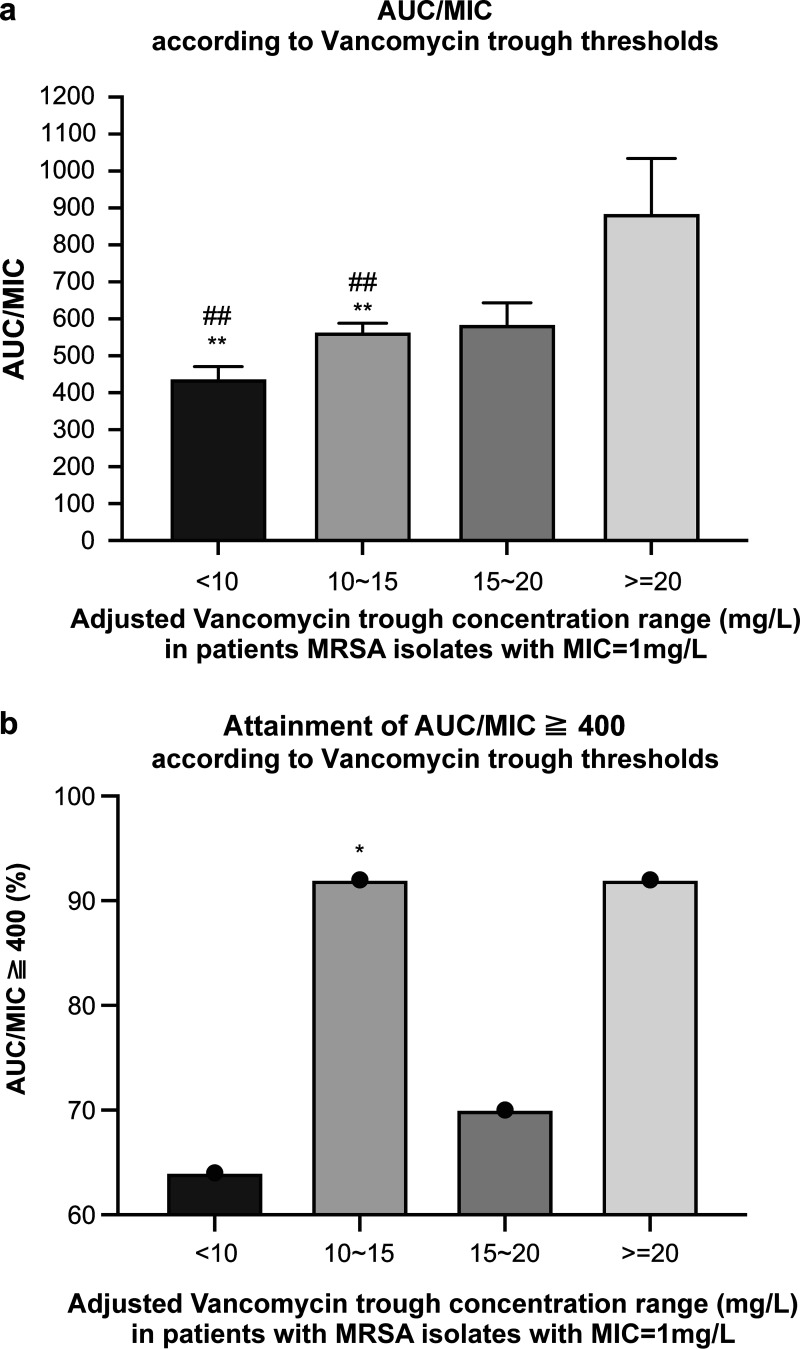
(a) Distribution of an AUC/MIC of ≥400 in the subgroup of 82 patients having MRSA isolates with an MIC = 1mg/L according to the four different adjusted vancomycin trough ranges. The Mann–Whitney U test (two-tailed) was performed for nonparametric data. Significant differences (*P* < 0.05) were observed between the groups. **, Indicates statistical significance when compared with the reference trough range of 10 to 15 mg/L in terms of attaining an AUC/MIC of ≥400. The Kruskal–Wallis test, followed by multiple comparison tests were used to analyze the data. The median values were significant (*P* < 0.05). ^##^, Indicates statistical significance when compared with the reference trough range of 10 to 15 mg/L in terms of attaining an AUC/MIC of ≥400. (b) Attainment of an AUC/MIC of ≥400 distribution according to the four different adjusted vancomycin trough ranges in patients with MRSA isolates with an MIC = 1 mg/L. Patients with troughs of 10 mg/L were less likely to attain an AUC/MIC of ≥400 compared with those with higher trough values (*P* < 0.05) *, Indicates statistical significance when compared to the reference trough range of 10 to 15 mg/L in terms of attaining an AUC/MIC of ≥400. AUC, area under the concentration–time curve; MIC, minimum inhibitory concentration.

Of the 102 patients, the MRSA strains isolated from 82 patients had an MIC of vancomycin = 1 mg/L. The demographic and vancomycin dosage characteristics of these patients, such as the dosage, MIC value, and derived AUC/MIC, were analyzed and are presented in Table 3. As represented in Figure 3a, a trough concentration of <10 mg/L was associated with the decreased likelihood of attaining AUC/MIC of ≥400. (Fig. 3a).

**TABLE 3:** Baseline demographics and adjusted vancomycin dosing characteristics in the subgroup of MRSA isolates of MIC (vancomycin) = 1 mg/L^*a*^

Characteristic	Adjusted vancomycin daily dose MIC = 1 group (*n* = 82)	
Mean age, yrs (SD)	64.72 ± 18.87	
Sex (%) Male Female	49 (59.76) 33 (40.24)	
Mean wt, kg (IQR)	56.03 (47.78 to 63.33)	
Mean Scr, mg/dL (IQR)	0.9 (0.6 to 0.9)	
Mean ClCr, mL/min (IQR)	81.15 (44.25 to 91.81)	
Mean wt-based dose, mg/kg (SD)	13.85 ± 4.59	
Mean wt-based daily dose, mg/kg/day (SD)	31.47 ± 14.01	
Mean daily dose, mg/day (SD)	1767.65 ± 847.45	
Mean AUC, mg × hr/L (SD)	576.89 ± 290.76	
Mean AUC/MIC ratio (IQR)	576.89 (419.01 to 647.57)	
AUC/MIC (n)	< 400	≥ 400
Trough < 10 mg/L Trough 10–15 mg/L Trough 15–20 mg/L Trough > 20 mg/L	9 2 6 1	16 22 14 12

a AUC, area under the concentration–time curve; MIC, minimum inhibitory concentration; MRSA, Methicillin-resistant Staphylococcus aureus; SD, standard deviation; IQR, interquartile range; Scr, serum creatinine; ClCr, creatinine clearance.

Of the 82 patients (which is 80.39% of the total population), 64 attained an AUC/MIC of ≥400, and the adjusted vancomycin average AUC/MIC was found to be 576.89. Approximately 92% of patients attained an AUC/MIC of ≥400 in the 10 to 15 mg/L trough range, which was 22% higher than the percentage of those in the 15 to 20 mg/L trough range that attained the same AUC/MIC. Moreover, as shown in Figure 3b, the difference between the 10 to 15 mg/L and <10 mg/L trough groups was statistically significant (*P* < 0.05).

### Secondary outcome.

**FIG 4:**
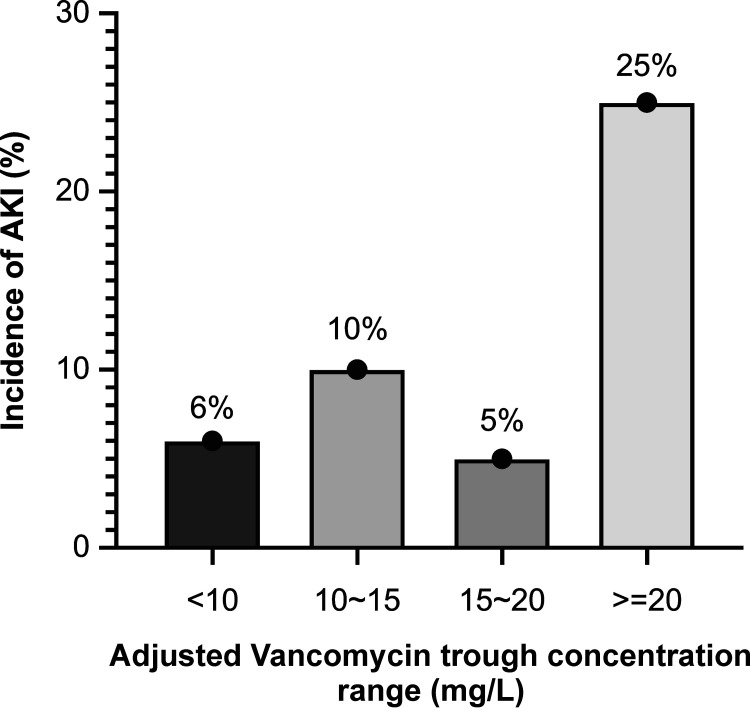
The incidence of AKI in the different vancomycin trough ranges (adjusted) groups. AKI, acute kidney injury.

While analyzing safety and clinical efficacy, no statistically significant difference (*P* = 0.16) in the correlation between the AUC/MIC ratio and the time required to attain the first negative culture or AKI (Tables 4 and 5) was observed. Patients with an AUC/MIC of ≥400 were more prone to AKI than patients with an AUC/MIC of <400, and the incidence was higher by 11% to 12% (11.94%:5.71% and 12.5%:0%; Table 4 and 5). Based on analyses in different trough ranges, the incidence of AKI in the >20 mg/L vancomycin trough range was the highest (Fig. 4).

**TABLE 4:** Adjusted AUC/MIC versus AKI and time to first negative culture in the total study population (*n* = 102)

Total (adjusted)	AUC/MIC ≧ 400 (*n* = 67)	AUC/MIC < 400 (*n* = 35)
Time to first (–) culture day, (±SD) patient no. (*n*)	8.2 (± 5.6) (*n* = 34)	6.6 (± 5.3) (*n* = 16)
AKI, *n* (%) Patient no. (*n*)	8 (11.94%) (*n* = 67)	2 (5.71%) (*n* = 35)

**TABLE 5:** Adjusted AUC/MIC versus AKI and time to first negative culture in the MRSA study population (*n* = 82) with MIC = 1mg/L^*a*^

MIC = 1 (adjusted)	AUC/MIC ≧ 400 (*n* = 64)	AUC/MIC < 400 (*n* = 18)
Time to first (–) culture day, (±SD) patient no. (*n*)	8.5 (±5.7) (*n* = 33)	9.0 (±6.4) (*n* = 5)
AKI, *n* (%) Patient no. (*n*)	8 (12.5%) (*n* = 64)	0 (0%) (*n* = 18)

a AUC, area under the concentration–time curve; MIC, minimum inhibitory concentration; AKI, acute kidney injury; SD, standard deviation.

## DISCUSSION

The relationship between the trough concentration and AUC/MIC as well as the correlation of trough concentration or AUC/MIC with renal toxicity and clinical efficacy were analyzed in this retrospective study. Our study results indicated that the percentage of patients in the 15 to 20 mg/L vancomycin trough range attaining AUC/MIC of ≥400 was not higher than the percentage of those in the 10 to 15 mg/L range (76%:67%). In a predominantly Asian neonatal study, an AUC/MIC of ≥400 could be attained using trough concentrations of 8 to 8.9 mg/L in >90% of the study population (9). Previously, Frymoyer et al. conducted another pharmacokinetic study on vancomycin in the neonatal population using a similar methodology. The study results indicated that a trough concentration of around 10 mg/L would be enough to ensure sufficient vancomycin exposure, which is acceptable for the pediatric population (9, 10). In a retrospective cohort study by Neely et al. in 2014, adults with vancomycin MIC = 1 mg/L for MRSA maintaining serum trough concentration of <15 mg/L could attain a therapeutic AUC/MIC of ≥400 (11). These findings are consistent with our study results.

In terms of side effects, a recent retrospective comparison of trough-guided versus AUC-guided vancomycin dosing in approximately 1,300 adults across four hospitals within the Detroit Medical Center was conducted. The study found that vancomycin therapy using AUCs can both preserve efficacy and reduce nephrotoxicity (11). In our study, the incidence of AKI was higher when vancomycin trough concentration was higher than 15 mg/L, especially when the trough was >20 mg/L with concomitant use of nephrotoxic medications. In terms of efficacy, there is insufficient data for a positive correlation with bacterial eradication. Owing to the clinical use of vancomycin therapy, routine cultures to confirm bacterial eradication may not be performed, except in cases of bacteremia or infective endocarditis (12). Our study did not find any significant difference in the time required for bacterial eradication, and we speculate that differences in time for bacterial eradication cannot be precisely analyzed because there is no fixed time for specimen collection. Moreover, there is no specimen collection in some clinical practice. Therefore, further studies in this field are warranted.

We provided key insights to clinicians that AUC/MIC of ≥400 was attained when vancomycin trough concentration was maintained at 10 to 15 mg/L, which significantly lowered the incidence of vancomycin-associated renal toxicity. In the future, guidelines on AUC/MIC-guided treatment with vancomycin can be revised for patients infected with MRSA strains with vancomycin MIC = 1 mg/L so that better efficacy and safety evaluations can be performed in clinical practice in Taiwan.

This study has certain limitations. For example, the sample size obtained during 2 years is small, and the interval between blood collections for trough measurement and the interval between specimen collections were inconsistent. Moreover, the currently used AUC formula may have underestimated the real AUC value, and the concomitant use of nephrotoxic medications was not discussed in the analysis of AKI. We hope that relevant future studies can overcome these limitations and offer optimized analyses.

## MATERIALS AND METHODS

### Study design and population.

In this pharmacokinetic study, data were collected by retrospectively reviewing the medical records of patients admitted to the Taipei Medical University Hospital (TMUH) who received intravenous vancomycin between January 2016 and December 2017. Inclusion criteria included hospitalized adult patients (aged 20–95 years) treated with vancomycin for MRSA infections, and having their serum drug concentration monitored and dosage adjusted by pharmacists. Patients were excluded if they had excessive fluctuations in renal function (defined as a difference of >0.2 mg/dL in the level of serum creatinine), were undergoing dialysis, or had their blood collected at an inappropriate time. The dosage of vancomycin was calculated based on the weight, and true trough concentrations were adjusted based on the time of blood collection and serum drug concentration. The AUC/MIC ratio was calculated based on the AUC/MIC calibration equation provided by DeRyke (13) in 2009 to analyze the correlation of trough concentration or AUC/MIC ratio with renal toxicity and clinical efficacy. The study was approved by the TMUH institutional review board prior to the initiation of the study.

### Data collection.

Tables represent the relevant collected patient data, including basic patient data, ward type, serum creatinine levels, site of bacterial culture, bacterial strains, initial and adjusted doses of vancomycin, number of days for which vancomycin therapy was administered, trough concentrations, MIC value, and the concomitant use of nephrotoxic medications.

### Calculations and definitions.

The Cockcroft–Gault equation was adopted to evaluate renal function: ClCr = ((140 − age) × weight)/(72 × Scr) (× 0.85 if female) (Scr = 0.8 if age >65) (14). AKI is defined as variations of over 0.5 mg/dL between consecutive serum creatinine measurements or an increase of 50% in serum creatinine level compared with the pre-dose level. If the actual body weight (ABW) is less than the ideal body weight (IBW), then ABW measurement is inserted into the equation; if ABW is greater than IBW but ABW is <130% of IBW, then IBW is entered into the equation; if ABW is >130% IBW, then the adjusted ABW is entered into the equation. Relevant equations are as follows: IBW: male = 50 + 2.3 [height (inch) − 60] and female = 45.5 + 2.3 [height (inch) − 60]; adjusted ABW: IBW + 0.4 (ABW − IBW).

**FIG 5:**
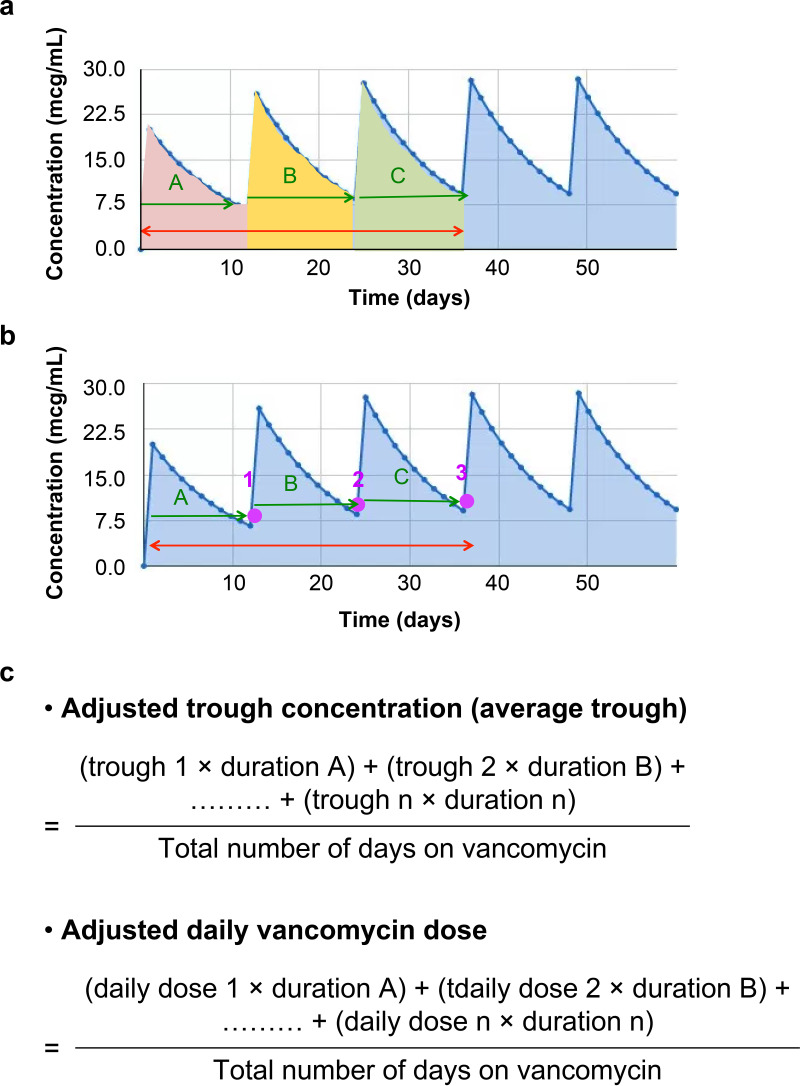
(a) Adjusted (average) vancomycin daily dose. (b) Adjusted (average) vancomycin trough. (c) The calibration equation: Vancomycin AUC = total average daily dose (mg)/vancomycin clearance (L/h). Vancomycin clearance = ClCr (mL/min) × 0.75 × 0.06 (L/h). AUC, area under the concentration–time curve; ClCr, creatinine clearance rate.

Considering additional fluctuations in drug concentrations in the human body, if the concentration measurements at each instance of blood collection are considered for calculations of individual AUCs in the same patient, the actual drug concentration in the same patient might not be accurately represented. Hence, average vancomycin daily dose (Fig. 5a) was used as the adjusted vancomycin daily dose to calculate the AUC. Further, the adjusted (average) vancomycin trough was calculated (Fig. 5b). The calibration equation is shown in (Fig. 5c).

### Outcome analysis.

The primary outcome was to determine whether there is an association between attaining a vancomycin trough within a specified range and reaching a calculated vancomycin AUC/MIC of ≥400. Patients were classified based on their troughs in the following ranges: <10 mg/L, 10 to 14.9 mg/L, 15 to 20 mg/L, and >20 mg/L. Trough concentrations were based on the adjusted vancomycin doses and trough concentrations. Attainment of the calculated target AUC/MIC of ≥400 was compared among the groups to analyze the percentage of patients attaining an AUC/MIC of ≥400 in each trough range.

The secondary outcome was to determine the corrected average vancomycin trough associated with the development of AKI, compare and correlate the mean predicted AUC/MIC and trough concentrations between patients who attained different trough concentrations in each trough range, and assess the time required to attain the first negative culture after treatment.

### Statistical analysis.

Descriptive statistics were used to determine the primary objective, and two-way Kruskal–Wallis test, followed by multiple comparison tests were performed for AUC comparisons among the different trough concentration groups. Only the statistically significant values are presented in the figures. Apart from the Kruskal–Wallis test, Mann–Whitney U test was performed to compare the recommended 10 to 15 mg/L group with the 10 mg/L group. Significant differences between the groups, indicated by *p* < 0.005, were determined. The analyses were performed on GraphPad prism (GraphPad prism version 9, version 9.3.1 for Windows, Inc., San Diego, CA, USA).
